# Prenatal detection and molecular cytogenetic characterization of 19q13.42 microduplication: three reported cases and literature review

**DOI:** 10.1186/s13039-020-00527-w

**Published:** 2021-01-15

**Authors:** Xinyue Zhang, Fagui Yue, Qingyang Shi, Yuting Jiang, Jing He, Leilei Li, Ruizhi Liu

**Affiliations:** 1 Center for Reproductive Medicine, Center for Prenatal Diagnosis, First Hospital, Jilin University, Changchun, 130021 China; 2 Jilin Engineering Research Center for Reproductive Medicine and Genetics, Jilin University, Changchun, 130021 China

**Keywords:** 19q13.42 mciroduplication, Chromosomal microarray analysis, Prenatal phenotypes, Follow up

## Abstract

**Background:**

Trisomy 19q is a recognizable syndrome and associated with a wide spectrum of clinical phenotypes in clinic. The purpose of this study was to explore the prenatal phenotypes of 19q13.42 duplication, which was rarely reported in clinic.

**Case presentation:**

Three pregnant women presenting diverse indications for prenatal diagnosis accepted amniocentesis: increased nuchal translucency and fetal pyelic separation (case 2) and high risk of maternal serum screening for Down syndrome (case 1 and case 3). Case 1 and case 2 shared similar duplicated locus in the region of 19q13.42, encompassing part *NLRP12* gene. The latter inherited the chromosomal duplication from the mother with normal phenotypes. Case 3 carried a 1.445 Mb duplication in the 19q13.42q13.43 region. It was proposed that evolutionary duplication of *NLRP12* gene could have a causative role in autoinflammatory diseases development. The genotype–phenotype correlation depends mainly on the duplicated size and functional genes involved, which is still yet to be determined. All pregnant women chose to continue the pregnancy and delivered healthy children with no apparent abnormalities.

**Conclusions:**

The 19q13.42 microduplications in our study were the smallest fragments compared to previous literature. Our findings enriched the prenatal phenotypes for this chromosomal microscopic imbalance. It was proposed that long term follow up analysis should be guaranteed till adulthood to determine whether there will be other emerging clinical symptoms and developmental-behavioral disorders for such carriers.

## Background

Chromosomal duplications are regarded to have close association with intellectual disability, growth retardation and other genetic disorders. Trisomy 19q, partial or entire duplication of long arm of chromosome 19, was a rare chromosomal anomaly which was first reported in 1976 [1]. Currently, the incidence rate was not clear in clinic. The clinical features of trisomy 19 described in previously reported patients were various and included growth retardation, developmental delay, intellectual disability, microcephaly, heart malformations, anomalies of the genito-urinary tract and/or the gastrointestinal system and seizures, which usually led to poor prognosis [2–5].

The formation mechanism of trisomy 19q could be due to the presence of a de novo duplication, parental balanced reciprocal translocation, pericentric inversion, or small supernumerary marker, which made it difficult to define a distinct genotype–phenotype correlation [6–8].

For prenatal cases presenting normal karyotypes, chromosomal submicroscopic imbalances of clinic significance could be diagnosed in approximately 1% of structurally normal pregnancies and 6% with structural malformations. Most frequent copy number variants (CNVs) observed in fetuses without structural anomalies included 15q11.2, Xp22.3, Xp21.1, 16p11.2, 1q21.1, 17p12, 16p13.11 and 22q11.21 [9]. To our knowledge, the first case of dup(19q) detected by prenatal diagnosis was reported in 1997 [3]. Herein, we describe three prenatal cases involving 19q13.42 microduplications presenting ultrasound anomalies or not, which are the smallest fragments compared to previous trisomy 19q cases.

## Case presentation

### Participants and clinical data

Three pregnant women underwent amniocentesis for cytogenetic and chromosomal microarray analysis (CMA) due to various indications for prenatal diagnosis: increased nuchal translucency and fetal pyelic separation (case 2) and high risk of maternal serum screening for Down syndrome (case 1 and case 3). All couples were nonconsanguineous and healthy. No family history of diabetes mellitus or congenital malformations were observed. All pregnant women denied any exposure to alcohol, teratogenic agents, irradiation, or infectious diseases during their pregnancies. This study protocol was approved by the Ethics Committee of the First Hospital of Jilin University (No. 2019-299), and written informed consents were obtained from all couples for publication of this case report and accompanying images.

## Methods

### Cytogenetic analysis

Amniocentesis was performed for karyotyping analysis with informed consent. Cytogenetic studies were performed on metaphases collected from cultured amniotic fluid cells from three pregnant women. Routine chromosome analysis was performed on G-banding techniques at 300–400 banding resolution prepared from the cultured amniotic fluid cells according to standard protocols. Twenty metaphases were analyzed for all samples. The International System for Human Cytogenetic Nomenclature (ISCN 2016) was used to describe the karyotype [10].

### Chromosomal microarray analysis

Genomic DNA was isolated from 10 mL amniotic fluid cells from all three pregnant women. The CytoScan 750 K array (Affymetrix, Santa Clara, CA, USA) was applied to detect the known and novel chromosomal CNVs across the entire genome following the manufacturer’s protocols and our previous study [11]. The procedures included genomic DNA extraction, digestion and ligation, PCR amplification, PCR product purification, quantification and fragmentation, labeling, array hybridization, washing and scanning. The CNVs detected were totally assessed by comparing them with published literature and the public databases: (1) Database of Genomic Variants (DGV) (http://dgv.tcag.ca/dgv/app/home), (2) DECIPHER (http://decipher.sanger.ac.uk/), (3) ISCA (https://www.iscaconsortium.org/) and (4) OMIM (http://www.ncbi.nlm.nih.gov/omim). Genomic positions refer to the Human Genome February 2009 assembly (GRCh37/hg19).

## Results

### Case 1

A 30-year-old, gravida 1, para 0, abortus 2, pregnant woman underwent amniocentesis for cytogenetic analysis and CMA detection due to the high risk of maternal serum screening for Down syndrome. No ultrasound findings were observed at 18 weeks of gestation. G-banding analysis showed that the karyotype of the fetus was 46,XY, but CMA revealed a 147 kb duplication in the region of 19q13.42. In order to identify whether the microduplication was de novo or parentally inherited, the couple accepted CMA after informed consents. It turned out that case 1 inherited the 19q microduplication from the mother with normal phenotypes. The couple chose to continue the pregnancy according to genetic counseling and delivered a male infant at 38w + 3d gestation, whose birth weight was 3300 g and length was 50 cm.

### Case 2

A 26-year-old, gravida 1, para 0, pregnant woman underwent ultrasound examination at 12 weeks and 26 weeks of gestation, which presented increased nuchal translucency and fetal pyelic separation in the fetus, separately. Afterwards, the woman underwent amniocentesis for cytogenetic analysis and CMA detection. The karyotype of the fetus was identified as 46,XY. However, CMA detected a 157 kb duplication in the region of 19q13.42. The couple declined to accept CMA to testify the origin of the 19q duplication and continued the pregnancy. The pregnant woman finally delivered a male infant at 38w + 6d gestation, whose birth weight was 3800 g and length was 50 cm.

### Case 3

A 29-year-old, gravida 1, para 0, pregnant woman underwent amniocentesis for cytogenetic analysis and CMA detection due to the high risk of maternal serum screening for Down syndrome. No ultrasound findings were observed at 21 weeks of gestation. The karyotype of the fetus was 46,XX. Then the CMA detected a 1.445 Mb duplication in the region of 19q13.42q13.43. The couple refused the CMA to confirm the chromosomal origin of the fetus and continued the pregnancy. The pregnant woman finally delivered a female infant at 39w + 2d gestation, whose birth weight was 3300 g and length was 50 cm.

We made a follow up on the postnatal health conditions for all cases, including congenital defects, developmental retardation, body stature, craniofacial dysmorphisms, and skeletal anomalies. All children were in healthy conditions, and no apparent abnormalities were observed till now, but long term follow up analysis was still necessary.

## Discussion and conclusions

In our study, we described three rare prenatal cases with pure 19q microduplications involving 19q13.42, ranging from 147 kb to 1.445 Mb. Only in case 2 was possible to study the origin of the anomaly and was proved to be inherited from the healthy mother. Currently, there is a lack of prenatal manifestations about this chromosomal microscopic imbalance. To the best of our knowledge, just five cases with 19q duplication were prenatally detected, presenting prenatal phenotypes including heart defect, anomalies of urinary tract, abnormal nuchal translucency/fold, intrauterine growth restriction and so on [3, 4, 12–14]. Only one case was involved in 19q microduplication [14]. In our study, the prenatally detected 19q duplicated loci were different from the cases mentioned above.

Trisomy 19q, could be regarded as a recognizable syndrome and associated with a wide spectrum of clinical phenotypes, including growth and psychomotor retardation, intellectual disability, low birth weight, microcephaly, short neck, heart malformations, skeletal anomalies, genitourinary anomalies, gastrointestinal defects, seizures and facial dysmorphisms (receding forehead, ptosis, hypertelorism, flat nasal bridge, small nose, short philtrum, down turned mouth, ear anomalies) [4, 12, 15]. Most trisomy 19q cases also carry monosomy of another chromosome, which makes it difficult to establish a clear phenotype-genotype correlation. Till now, only 19q12-q13.2 duplication is recognized as an obesity-related syndrome with intellectual disability and minor facial findings [16].

Pure 19q duplications, as a rare chromosomal anomaly, can be usually discovered in live-borns by molecular genetic technique [17]. Till now, there have been limited reports on comparable 19q13.42q13.43 microduplications. To better interpret the genotype–phenotype correlation of this region, we made a summary on clinic data in postnatal and prenatal cases with pure 19q13.42q13.43 duplication, as shown in Table 1 [14, 18–20]. The age of these cases ranged from neonate to 8 years. All 19q microduplications were identified through molecular cytogenetic techniques, which varied in size, ranging from 25 kb to 1.445 Mb. Among these duplications, 2/7 cases were de novo, 3/7 cases were parentally inherited, and 2/7 cases were not available. 3/7 cases were postnatally confirmed. Among them, rare anomalies, such as systemic-onset juvenile idiopathic arthritis, Duane retraction syndrome type III and autism spectrum disorder, were observed. 4/7 cases were prenatally detected. Two cases presented abnormal ultrasound findings, including intrauterine growth restriction, fetal pyelic separation and increased nuchal translucency. And the other two cases showed no ultrasound anomalies during the pregnancies. It seemed that the prenatal trisomy 19q13.42q13.43 cases might exhibit normal or abnormal ultrasound findings. Although the infants (cases 1 to 3) are now in healthy state, long term follow up analysis is still necessary to confirm whether there will be other emerging clinical symptoms or developmental-behavioral disorders. Among the 3/7 cases with parental inheritance, no abnormal parental phenotypes were observed. The incomplete penetrance might explain why the 19q duplications were occasionally transmitted through unaffected parents. In addition, these 19q13.42q13.43 duplications encompassed different functional genes, which probably explained the phenotypic diversities in these patients. Generally speaking, the clinic presentations of 19q13.42q13.43 duplication were diverse and atypical, and more evidence should be accumulated to investigate the genotype–phenotype correlation.

**Table 1:** Clinical features of previously published cases involving pure 19q13.42q13.43 duplication

References	Tadaki et al. [18]	Abu-Amero et al. [19]	Pinto et al. [20]	Petre et al. [14]	Our case 1	Our case 2	Our case 3
Age/sex	N.A	8y/F	N.A./F	26w/N.A	7 m/M	5 m/M	1 m/F
Duplicated region	19q13.42	19q13.42	19q13.42	19q13.42	19q13.42	19q13.42	19q13.42q13.43
Duplicated size	77 kb 622 kb	95 Kb	25 kb	1.06 Mb	147 kb	157 kb	1.445 Mb
Inheritance	De novo	De novo	Maternal	Paternal	Maternal	N.A	N.A
Gestational age	N.A	N.A	N.A	N.A	38w + 3d	38w + 6d	39w + 2d
Weight at birth	N.A	N.A	N.A	N.A	3.3 kg	3.8 kg	3.3 kg
Length at birth	N.A	N.A	N.A	N.A	50 cm	50 cm	50 cm
Karyotype	N.A	N.A	N.A	N.A	46,XY	46,XY	46,XX
Postnatal/prenatal	Postnatal	Postnatal	Postnatal	Prenatal	Prenatal	Prenatal	Prenatal
Chromosomal microarray results (hg19)	19q13.42(55498558–55575815) × 3 19q13.42(55685280–56299373) × 3	19q13.42(66234676–6329930) × 3	19q13.42(55153092–55178778) × 3	19q13.42(53759522–54821015) × 3	19q13.42(54175437–54321990) × 3	19q13.42(54165218–54321990) × 3	19q13.42q13.43(56124077–57568700) × 3
Referred critical gene	NLRP2, NLRP9, NLRP11, IL11, HSPBP1	KIR2DL4, KIR3DP1, KIR2DL1, KIR3DL3	LILRB4, LIR5	ZNF331	NLRP12	NLRP12	NLRP4 NLRP5 NLRP8, -9, -11, -13 ZNF580, -444, -582, -667, -71
Parental phenotype	N.A	N.A	Healthy mother	Healthy father	Healthy parents	Healthy mother	Healthy parents
Clinical manifestation	Systemic-onset juvenile idiopathic arthritis	Prominent forehead, low-set ears, clinodactyly, Duane retraction syndrome type III, history of infections	Language disorders, ASD, no epilepsy, no dysmorphic features	G1P0, Intrauterine growth restriction, intrauterine fetal death	G4P1A2, DS:1/205, ultrasound findings at 18 weeks of gestation showed no anomalies. No evident abnormalities after birth	G1P0, ultrasound findings: increased nuchal translucency at 12 weeks of gestation, fetal pyelic separation at 26 weeks of gestation. No evident abnormalities after birth	G1P0, DS:1/43, ultrasound findings at 21 weeks of gestation detected no anomalies. No evident abnormalities after birth

F, female; M, male; m, months; y, years; w, weeks; +, present; –, not present; N.A., not available; Gravida, G; Para, P; Abortus, A; DS, Down’s syndrome; ASD, Autism Spectrum Disorder

**Fig. 1:**
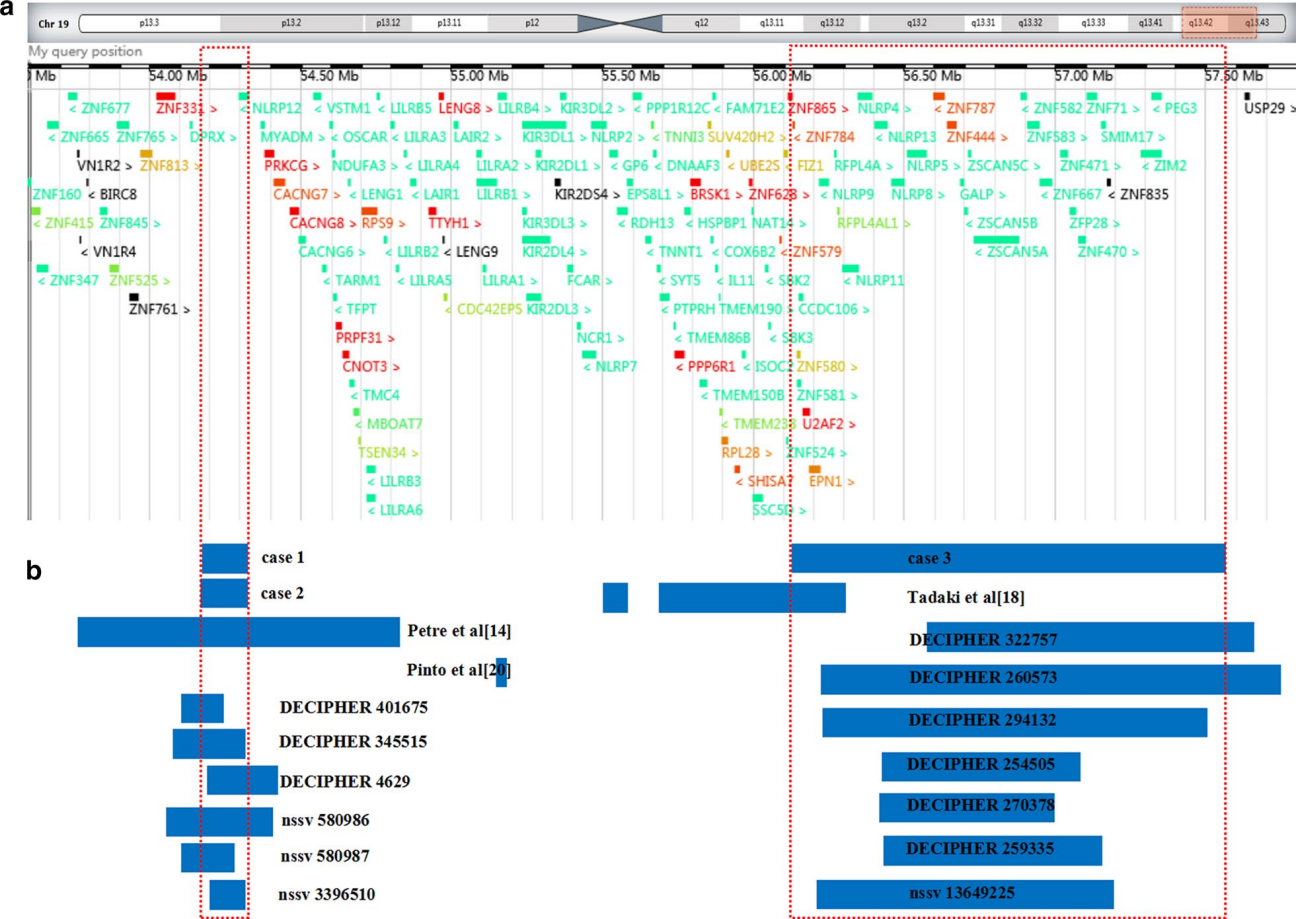
Scale representation of the duplicated region in the long arm of chromosome 19q13.42q13.43 (https://decipher.sanger.ac.uk/). **a** Location of genes in the region. **b** Microduplications detected in the present cases (cases 1 to 3) and previously reported microduplications involving 19q13.42q13.43 in the literature and public databases

In addition, we also made detailed comparisons of cases harboring 19q13.42 microduplication in DECIPHER and ISCA databases to elaborate diverse clinical phenotypes (Fig. 1). Six cases overlapping similar duplications with cases 1 and 2 were recorded. The proportions of pathogenicity were as follows: likely pathogenic (1/6) and uncertain (5/6). The incidence rate of clinical features was as follows: Development delay (3/6), intellectual disability (3/6), and autism (2/6). Seven cases shared similar duplication with case 3 in the databases. The clinic pathogenicity in this duplicated locus was uncertain. Only one patient nssv 13649225 presented failure to thrive, hemolytic anemia, short stature and spherocytosis, and no clinic phenotypes were observed for the other six cases. Generally speaking, more evidence should be accumulated to explore the pathogenicity of 19q13.42 microduplication.

The CNVs detected in our case 1 and case 2 shared similar 19q13.42 microduplication, encompassing part *NLRP12* gene (OMIM 609648; chr19: 54165218–54321990). *NLRP12* gene, also known as *RNO*, *PYPAF7*, and Monarch-1, encodes the protein of NLRP superfamily, which is implicated in the activation of proinflammatory caspases and hyperproduction of interleukin-1β [21]. *NLRP12* plays critical roles in the regulation of NF-κB signaling, inflammasome activation, dendritic cell migration, and transcription of MHC class I genes [22]. The mutations in *NLRP12* have been associated with familial cold autoinflammatory syndrome-2 (FCAS2; OMIM 611762), which displays autosomal dominant inheritance. This syndrome can be induced after exposure to cold and characterized by skin urticaria, arthralgia, conjunctivitis, musculoskeletal symptoms, deafness, lymphadenopathy, and abdominal pain, most of which are accompanied by recurrent fever and serologic evidence of inflammation [23, 24]. As is known, dosage-sensitive genes with genome alteration could result in phenotypic effects and be associated with human diseases, including heart disease, cancers, diabetes, neuropsychiatric disorders and others [25]. According to the literature review and database searching, there is no available pathogenic evidence for triplosensitivity associated with *NLRP12*. However, Galozzi et al. [24] proposed that evolutionary duplication of this gene can have a causative role in autoinflammatory diseases development. Hence, we suggested that these two infants should be followed up regularly on growth and health conditions, especially for autoinflammatory diseases.

For case 3, the detected 19q13.42q13.43 duplication contains 19 OMIM genes. This locus comprises several NLRP (Nucleotide-binding oligomeriztion domain, Leucine rich Repeat and Pyrin domain) family members. *NLRP4* is mainly responsible for the inhibition of NF-κB signaling, negative regulation of RLR signaling, autophagy inhibition. *NLRP5* is related to the regulation of caspase activation, apoptosis in injured neurons, and embryonic development. The functions of other members (*NLRP8, -9, -11, -13*) are still to be further investigated [18, 22]. As is known, the zinc finger proteins (ZNFs) are the largest transcription factor family in human genome, which contain finger-like protrusions and play critical roles in physiological and pathophysiological mechanisms [26]. Previous research showed that duplications of zinc finger genes commonly occurred during the evolution [27]. The duplicated locus also included *ZNF580, -444, -582, -667, -71*. Research on the functions of these genes are rare. According to OMIM database, the variations in *ZNF582* gene might be associated with intellectual disability. *ZNF580* is supposed to be involved in endothelial cell proliferation and migration. Enhancing expression of *ZNF667* can inhibit the apoptosis via inhibiting Bax and Fas expression. And *ZNF667* might be a new oncogene in human hepatocellular carcinoma as a new therapeutic target through enhancing *BCL-2* and decreasing *BAX* expression. Till now, the duplication of these genes could hardly result in severe outcomes in clinic.

Since the young ages of our subjects and the lacking of comparable individuals limit the assessment of growth and development in future, long-term follow up analysis should be guaranteed. In addition, phenotypic diversity, incomplete penetrance, and the inheritance might also be associated with the clinic pathogenicity of 19q microduplication to different degrees, and the clinic data should be further collected.

In our study, we described three rare prenatal cases consisting of 19q microduplication, encompassing 19q13.42 locus. Our findings would enrich the phenotypic spectrums of 19q duplication. It is believed that more trisomy 19q cases with clinic manifestations and molecular genetic characterization would further help to refine clear genotype–phenotype correlations. For prenatally detected 19q duplications without evident abnormalities, long term follow up should be guaranteed to confirm whether there will be other clinical symptoms or developmental-behavioral disorders afterwards.

## Data Availability

The data and material used or analysed during the current study are available from the corresponding author on reasonable request.

## Abbreviations

CMA: Chromosomal microarray analysis
CNVs: Copy number variants
DGV: Database of Genomic Variants
ISCN 2016: International System for Human Cytogenetic Nomenclature
NCBI: National Center for Biotechnology Information
OMIM: Online Mendelian Inheritance in Man
